# The Impact of Night Shift Work on Mental Health Outcomes in a Tunisian Industrial Setting

**DOI:** 10.1192/j.eurpsy.2025.1578

**Published:** 2025-08-26

**Authors:** A. Chouchane, Y. Tebourbi, M. Bouhoula, I. Kacem, R. Ghammem, K. Hadj Mabrouk, I. Kanoun, B. Zendah, A. Aloui, M. Maoua, A. Brahem, H. Kalboussi, S. Mhamdi, L. Bouzgarrou, S. Chatti, O. ElMaalel

**Affiliations:** 1 Occupational Medicine and Professional Pathologies Department, Farhat Hached Teaching Hospital, Tunisia, University of Sousse, Faculty of Medicine of Sousse, Sousse, Tunisia; 2 3- Service d’épidémiologie, CHU Farhat Hached Sousse, Tunisie; 3 Inter-company occupational health services of the Governorate of Sousse, Tunisia, Sousse; 4 Service d’épidémiologie, CHU Fattouma Bourguiba Monastir, Faculté de Médecine de Monastir, Tunisie; 5 Service de Médecine du Travail, Hôpital Haj Ali Soua Ksar Hellal, Faculté de Médecine de Monastir, Tunisie, Monastir, Tunisia

## Abstract

**Introduction:**

The increasing prevalence of shift work, particularly night shifts, in modern industrial settings has raised concerns about its potential detrimental effects on workers’ health. Disruptions to circadian rhythms, sleep deprivation, and social isolation associated with night shift work have been linked to a range of physical and mental health problems.

**Objectives:**

This study aims to investigate the specific impact of night shift work on the mental health outcomes of Tunisian industrial workers.

**Methods:**

This is a cross-sectional study carried out during 3 years among active workers working in the inter-company occupational health services of Sousse. All participants had a fixed night work schedule. Data collection was based on a pre-established anonymous questionnaire. Job strain was assessed with Karazek questionnaire.

**Results:**

A total of 453 employees were included in our study. Mean age was 32.12 ± 7.68 years. Half of the participants were women (52%). Sixty percent of participants were not married. Tobacco consumption was identified among 26% of the participants. The most affected sector of activity was the electronic one (63%). The average occupational seniority was 7.78 ± 6.407. Job strain was revealed in 23.4% of employees. High psychological demand was noted among 46.5% of cases. Low job control was identified among 57,4% of participants. Low social support was noted among 68% of participants.

**Conclusions:**

These results underscore the need for targeted interventions to protect the health and well-being of night shift workers. Future research should explore the effectiveness of various strategies, such as scheduling modifications, workplace accommodations, and health promotion programs, in mitigating the negative impacts of night shift work.

**Disclosure of Interest:**

None Declared

